# Supplementary material for: Low birth weight and associated factors among HIV positive and negative mothers delivered in northwest Amhara region referral hospitals, Ethiopia,2020 a comparative crossectional study
Source: PLoS One. 2022 Feb 11;17(2):e0263812. doi: 10.1371/journal.pone.0263812 (PMC8836330; doi:10.1371/journal.pone.0263812)
Supplement: S1 File — (ZIP) [file pone.0263812.s001.zip › questioniare.docx]

# Data extraction shit for HIV positive

Name of the health hospital: ----------------------------------

A record number (code): -----------------------------------------

**Part 1 socio-demographic and behavioral variables**

| **Q. Number** | **Questions** | **Choice for response** | **Code** |
| --- | --- | --- | --- |
| **101** | Age of the mother at delivery | **……………..** |  |
| **102** | Religion | 1. Muslim  2. Orthodox  3. Protestant  4. Others |  |
| **103** | Residence | 1. Rural  2. Urban |  |
| **104** | Maternal Educational status | 1. Not read and write  2. Read and write  3. Primary education  4. Secondary education and  Above |  |
| **105** | Occupation | 1. Farmer  2. Merchant  3. Housewife  4. Government employed  5. If Other specify------- |  |
| **106** | Marital status | 1.Single  2. Married  3. Divorced  4. Widowed |  |
| **107** | History of substance use (including heavy alcohol drinking and smoking) | 1. Yes  2. No |  |

**Part 2 medical related factor**

| **Q. No** | **Questions** | **Choice for response** | **Code** |
| --- | --- | --- | --- |
| **201** | mother’s hemoglobin level during pregnancy | ……………. |  |
| **202** | known history chronic hypertension | 1. Yes  2. No |  |
| **203** | known history of Diabetes Mellitus | 1. Yes  2. No |  |
| **204** | STI during the current  pregnancy which is confirmed | 1. Yes  2. No |  |
| **205** | UTI during the current pregnancy which is confirmed | 1. Yes  2. No |  |
| **206** | RH factor | 1.positive  2.negative |  |

**Part 3 past and current obstetric related factor**

| **No** | **Questions** | **Choice for response** | **Code** |
| --- | --- | --- | --- |
| **301** | Gravidity | ……………… |  |
| **302** | Number of previous birth(parity) | ………………. |  |
| **303** | History of LBW | 1.yes  2.no |  |
| **304** | History of abortion | 1.yes  2.no |  |
| **305** | History of still birth or neonatal loss | 1.yes  2.no |  |
| **306** | APH during current pregnancy | 1.yes  2.no |  |
| **307** | PROM during current pregnancy | 1.yes  2.no |  |
| **308** | PIH during pregnancy | 1.yes  2.no |  |
| **309** | Presence of ANC follow up | 1.yes  2.no |  |
| **310** | Number of ANC follow up | ……………… |  |
| **311** | Time to initiate ANC follow up | 1.first trimester  2. second trimester  3. third trimester |  |
| **312** | Malpresentation | 1.yes  2.no |  |
| **313** | Labor status | 1.induced  2. spontaneous |  |
| **314** | Prolonged labor | 1.yes  2.no |  |
| **315** | Type of pregnancy | 1.single  2. multiple |  |
| **316** | Mode of delivery | 1.SVD  2.ceserian section  3. instrumental delivery |  |
| **317** | Pregnancy status | 1.planned and wanted  2. unplanned but wanted and supported  3.unplanned and unwanted but supported  4. unplanned, unwanted and unsupported |  |

**Part 4 Nutritional related factor**

| **Q. no** | **Questions** | **Choice of response** |  |
| --- | --- | --- | --- |
| **401** | Pre pregnancy BMI of the mother | **……………..** |  |
| **403** | MUAC of the mother | **…………….. Cm** |  |
| **404** | Iron/folate intake during pregnancy | 1.Yes  2.No |  |
| **405** | If yes for question no 405. how many months the mother takes Iron/folate | 1 less than 2 months  2.2-3 month  3. greater than 3 months |  |
| **406** | Nutritional counseling during ANC/PMTCT | 1.yes  2.no |  |

**Part 5 HIV disease status-related factor**

| **Q. No** | **Questions** | **Choice for response** |  |
| --- | --- | --- | --- |
| **501** | Known HIV/ AIDS status before the current pregnancy | 1.Yes  2.No |  |
| **502** | ARV intervention | 1.Yes  2.No | If no go to Q 506 |
| **503** | Types of ARV | 1.HAART  2. prophylaxis |  |
| **504** | Time HAART initiated | 1.before pregnancy  2.during pregnancy |  |
| **505** | Types of HAART regimen | …………………. |  |
| **506** | HAART adherence | 1.good  2.fair  3.poor |  |
| **507** | Presence of PMTCT follow up | 1.Yes  2.No |  |
| **508** | Time of initiation of PMTCT | 1.antepartum  2. post-partum |  |
| **509** | Maternal CD4 count during pregnancy | ……………cells/mm^3^ |  |
| **510** | viral load during pregnancy | ………………copies/ml |  |
| **51** | clinical stage of the mother during pregnancy | 1.stage 1  2. stage 2  3. stage 3  4. stage 4 |  |
|  |  |  |  |
|  |  |  |  |

**Part 6 new born characteristics**

| **Q. no** | **Questions** | **Choice for response** |  |
| --- | --- | --- | --- |
| **601** | Status of a newborn baby at birth | 1.live birth  2. stillbirth |  |
| **602** | Sex of newborn | 1.male  2. female |  |
| **603** | Gestational age at birth | ……………. Week |  |
| **604** | Birth weight | ………….. gram |  |

# Data extraction shit for HIV negative

Name of the health hospital: ----------------------------------

A record number (code): -----------------------------------------

**Part 1 socio-demographic and behavioral variables**

| **Q. Number** | **Questions** | **Choice for response** | **Code** |
| --- | --- | --- | --- |
| **101** | Age of the mother at delivery | **……………..** |  |
| **103** | **Residence** | 1. Rural  2. Urban |  |
| **104** | History of substance use (including heavy alcohol drinking and smoking) | 1. Yes  2. No |  |

**Part 2 medical related factor**

| **Q. No** | **Questions** | **Choice for response** | **Code** |
| --- | --- | --- | --- |
| **201** | mother’s hemoglobin level during pregnancy | …………….. g/dl |  |
| **202** | known history chronic hypertension | 1. Yes  2. No |  |
| **203** | known history of Diabetes Mellitus | 1. Yes  2. No |  |
| **204** | STI during the current  pregnancy which is confirmed | 1. Yes  2. No |  |
| **205** | UTI during the current pregnancy which is confirmed | 1. Yes  2. No |  |
| **206** | RH factor | 1.positive  2.negative |  |

**Obstetric related factor**

| **No** | **Questions** | **Choice for response** | **Code** |
| --- | --- | --- | --- |
| **301** | Gravidity | ………………… |  |
| **302** | Number of previous birth(parity) | …………………… |  |
| **303** | History of LBW | 1.yes  2.no |  |
| **304** | History of abortion | 1.yes  2.no |  |
| **305** | History of still birth or neonatal loss | 1.yes  2.no |  |
| **306** | APH during pregnancy | 1.yes  2.no |  |
| **307** | PROM during pregnancy | 1.yes  2.no |  |
| **308** | PIH during pregnancy | 1.yes  2.no |  |
| **309** | Presence of ANC follow up | 1.yes  2.no |  |
| **310** | Number of ANC visit |  |  |
| **311** | Time to initiate ANC visit | 1.first trimester  2. second trimester  3. third trimester |  |
| **312** | Malpresentation | 1.yes  2.no |  |
| **313** | Labor status | 1.induced  2. spontaneous |  |
| **314** | Prolonged labor | 1.yes  2.no |  |
| **315** | Type of pregnancy | 1.single  2. multiple |  |
| **316** | Mode of delivery | 1.SVD  2.ceserian section  3. instrumental delivery |  |
| **317** | Pregnancy status | 1.planned and wanted  2. unplanned but wanted and supported  3.unplanned and unwanted but supported  4. unplanned, unwanted and unsupported |  |

**Part 4 Nutritional related factor**

| **Q. no** | **Questions** | **Choice of response** |  |
| --- | --- | --- | --- |
| **401** | Iron/folate intake during pregnancy | 1.Yes  2.No |  |
| **402** | If yes for question no. how many months the mother takes Iron/folate | ……………………….. |  |
| **403** | Nutritional counseling during ANC | 1.yes  2.no |  |

**Part 5 new born characteristics**

| **Q. no** | **Questions** | **Choice for response** |  |
| --- | --- | --- | --- |
| **501** | Status of a newborn baby at birth | 1.live birth  2. stillbirth |  |
| **502** | Sex of newborn | 1.male  2. female |  |
| **503** | Gestational age at birth | …………. week |  |
| **504** | Birth weight | ……………… gram |  |
