# Supplementary material for: Low birth weight and associated factors among HIV positive and negative mothers delivered in northwest Amhara region referral hospitals, Ethiopia,2020 a comparative crossectional study
Source: PLoS One. 2022 Feb 11;17(2):e0263812. doi: 10.1371/journal.pone.0263812 (PMC8836330; doi:10.1371/journal.pone.0263812)
Supplement: Attachment — Submitted filename: Authors Response 2.docx [file pone.0263812.s003.docx]

**Authors’ response to concerns of Editor and Reviewers**

Dear Editor and Reviewers, we the authors of this article would be very happy to bear our sincere gratitude for your enormous contribution - rigorous editorial and review concerns for which we will go one by one to make our manuscript suitable for publication in PLOS ONE journal. Therefore, we are going to respond the Editor, Reviewer #1 concerns respectively as presented here under:

**Editor concerns and Authors’ responses**

**Editor concern**: “1. Please review your reference list to ensure that it is complete and correct. If you have cited papers that have been retracted, please include the rationale for doing so in the manuscript text, or remove these references and replace them with relevant current references. Any changes to the reference list should be mentioned in the rebuttal letter that accompanies your revised manuscript. If you need to cite a retracted article, indicate the article’s retracted status in the References list and also include a citation and full reference for the retraction notice.

**Authors’ response**: Dear Editor, we reviewed our Manuscript reference list; it is complete and correct and there is no retracted cited paper

**Reviewer #1 concerns and Authors’ responses**

**Reviewer concern 1:** There are still a few grammatical errors such as "is suffered from" LBW and 'Result' instead of results.

**Authors’ response**: Dear Reviewer, ‘is suffered from’ LBW and 'Result' instead of results is corrected as “suffered from” LBW and “Results” in the revised version of the manuscript with track changes and without track changes.

**Reviewer concern 2:** It was stated that chewing "chat" would be explained in the text as "khat (stimulant)" but this was not done

**Authors’ response**: Dear Reviewer, the word ‘chat’ in the original manuscript is corrected as “Khat (**stimulant drug)**” in the revised version of the manuscript with track changes and without track changes.
